# Reducing Visuospatial Pseudoneglect in Healthy Subjects by Active Video Gaming

**DOI:** 10.3390/brainsci13060877

**Published:** 2023-05-29

**Authors:** Giuditta Gambino, Lorenzo Pia, Giuseppe Ferraro, Filippo Brighina, Danila Di Majo, Fabrizio Di Giovanni, Tommaso Ciorli, Pierangelo Sardo, Giuseppe Giglia

**Affiliations:** 1 Department of Biomedicine, Neuroscience and Advanced Diagnostics (BiND), Section of Human Physiology, University of Palermo, 90134 Palermo, Italy; giuseppe.ferraro@unipa.it (G.F.); filippo.brighina@unipa.it (F.B.); danila.dimajo@unipa.it (D.D.M.); fabrizio.digiovanni@community.unipa.it (F.D.G.); pierangelo.sardo@unipa.it (P.S.); giuseppe.giglia@unipa.it (G.G.); 2 SAMBA—(SpAtial, Motor & Bodily Awareness) Psychology Department & Neuroscience Institute of Turin (NIT), University of Turin, 10123 Turin, Italy; lorenzo.pia@unito.it (L.P.); tommaso.ciorli@unito.it (T.C.); 3 Euro-Mediterranean Institute of Science and Technology (IEMEST), 90139 Palermo, Italy

**Keywords:** pseudoneglect, visuospatial attention, exergaming, video gaming

## Abstract

Pseudoneglect phenomenon refers to a condition in which healthy subjects tend to perceive the left side of exactly bisected lines as being slightly longer than the right one. However, behavioural data showed that athletes practising an open-skill sport display less pseudoneglect than the general population. Given the fact that so-called exergames (also known as active video games) are platforms designed to fully mimic sport activity, this work intends to investigate whether and how a one-week training period of exergame open-skill sport can determine a similar decrease in pseudoneglect. Fifteen healthy participants (non-athletes) responded to a visuospatial attention task and a control memory task in basal conditions (t0: Pre-game) and after a short period (one week, one hour/day) of tennis exergaming (t1: Post-game). In the Post-game condition, subjects from this experimental group (ExerGame group: EG) reduced leftward space overestimation and made significantly fewer leftward errors compared to the Pre-game condition. Additionally, two other experimental groups were employed: one evaluated within the same conditions of the main experiment but using a non-exergame (Non-Exergame groups: NEG) and the other one without any video game stimulus (Sedentary group: SE). Our findings suggest that daily training of a tennis exergame seems to be able to improve visuospatial attention isotropy by reducing leftward space overestimation, whereas outcomes from non-exergaming and sedentary activity do not modify subjects’ performance.

## 1. Introduction

In the field of sport sciences, spatial attention, i.e., the ability to orient to salient visual stimuli and to parse the visual world [1], has been considered an important determinant of success in open-skill sports [2], which feature environments in constant change where skills are externally paced and have to be continually adapted. These types of sports—including, for example, volleyball, basketball and tennis—have been recently described as those that require adaptability and quick decision-making [3]. The ability to process spatial information and perceive differences or asymmetries correctly in both hemi-visual fields allow athletes to successfully perform and rapidly react to incoming stimuli (the ball or an opponent feint, for example). In contrast, closed-skill sports (e.g., swimming, golf and running) are rather self-paced with a relatively stable environment [3]. Given the distinct characteristics of open- (e.g., volleyball) and closed-skill (e.g., rowing) sports, it has been hypothesised that professional athletes might show differences in their visuospatial attention abilities depending on the specific requirements of their form of sport. Indeed, a study found that professional volleyball players (highest level, Italian A league) were faster and more accurate on a visuospatial attention lateralisation task (Milner Landmark) compared with the general sedentary population and athletes practising a closed-skill sport [4]. Considering that this improved performance was specifically due to a reduced leftward bias in visuospatial attention, the authors speculated that open-skill sport players counteract the physiological imbalance between the hemispheres [5] underlying the pseudoneglect phenomenon. This refers to a weak but consistent bias of spatial attention towards the left side of space [6,7,8]. Functional asymmetries in cortical activity have been recently described due to impaired visual input [9,10]; however, there is compelling evidence for primary physiological and functional asymmetries between the left and right hemisphere in attentional control that are lateralised to the right hemisphere, as evidenced by neuroimaging [6] and non-invasive brain stimulation (NIBS) studies [11]. Lesion studies in stroke patients provide clear evidence that spatial neglect, a neurological syndrome characterised by severe attentional deficits, is more common after right hemisphere damage. Specifically, neglect patients show strong biases in the allocation of attention and might fail to perceive the contralesional side of space altogether [12].

At a behavioural level, this triggers an imbalance in the distribution of attention in the environment. Consequently, this leads to the tendency to perceive the left segment of an exactly bisected line to be slightly longer, which is typical of pseudoneglect [7], both in egocentric and allocentric space mapping [13,14,15,16]. While the existence of pseudoneglect is undisputed, the strength and even direction of the attention bias are influenced by various factors such as age, gender, handedness and stimulus material, as widely described in previous meta-analyses [13,17,18].

In recent years, many video game platforms provided the possibility to play so-called ‘exergames’, i.e., active video gaming requiring physical activity like a real sport [19]. Differently from traditional video gaming (in our work, also referred to as “non-exergame”), these types of platforms allow game control by recognizing the whole upper or lower limb function, balance and gait, rather than simple hand movements [20]. It has been suggested that exergames can contribute to a healthy lifestyle by increasing physical activity in children [21,22], adults [23,24] and neurologically impaired people [25,26,27] and influencing heart rate and oxygen uptake responses similarly to that of light and moderate exercise [28]. Exergames are also able to affect cognitive performance [29], which was also reflected by EEG power spectra changes [30], and this could provide new foundations for building a bridge over behavioural correlates and neuronal bases of higher processes [29,30,31].

Moreover, recent meta-analyses found exergames to exert beneficial effects on global cognition in adults with mild cognitive impairment (MCI) at least as well as aerobic exercises by increasing global cognition and, more specifically, executive functions, attentional processing and visuospatial skills [32,33]. On these bases, in the current study, it was hypothesised that presenting in an exergame platform a similar cognitively demanding environment that required better performance in visuospatial attention lateralisation in real open-skill sport would be able to modulate the same functions in non-athletes. To this aim, it was investigated for the first time whether changes in visuospatial attentional control could emerge from a short period of playing an exergame open-skill sport with an exergame platform. Therefore, a modified version of the Milner Landmark task [34,35] was applied in order to investigate whether visuospatial attentional differences could emerge after a short period of exergaming. To do so, the current study was structured with a visuospatial attention task through the modified Milner Landmark task and a stimulation task that were delivered to three experimental groups of participants. These subjects were, respectively, exposed to an exergaming open-skill sport, to non-exergaming and to a sedentary lifestyle, as described in detail in the experimental procedures and coherently commented in the results and discussion sections. Based on the findings on real sport described above, this research was designed to uncover if a similar reduction of pseudoneglect would be encountered after practising an open-skill sport exergame. The performance on a control memory task was expected to be unaffected, implying a specific improvement in visuospatial attention due to the exergame platform. Thus, this study could provide intriguing novel knowledge in the field of the pseudoneglect phenomenon. 

## 2. Materials and Methods

Detailed descriptions of the experimental procedures are presented in this section.

### 2.1. Participants

In the present experimental study, 45 right-handed, Caucasian, master’s degree medicine and surgery students (27 males; 18 females, aged 26 ± 2.5; years of education, 16–18) were enrolled for the protocol. All subjects had no evidence of brain dysfunction, as assessed by two clinical neurologists by means of general and neurological examination. The protocol was approved by the Biomedicine, Neuroscience and Advanced Diagnostics (BiND) Department scientific committee of the University of Palermo (code Palermo1_05_2021). All subjects had normal or corrected-to-normal visual acuity. Each subject declared themselves as sedentary, i.e., “in a condition of habitual lack of physical activity”. All subjects declared that they played video games habitually, but none had any previous experience with exergames. 

All participants were right-handed, as assessed by the Edinburgh Handedness Inventory [36]. 

They gave their informed consent in participating in the study. All data were collected anonymously. 

Participants were divided into three different experimental groups, each comprising 15 subjects: ExerGame (EG), Non-Exergame (NEG) and Sedentary (SE).

### 2.2. Tasks

**Figure 1:**
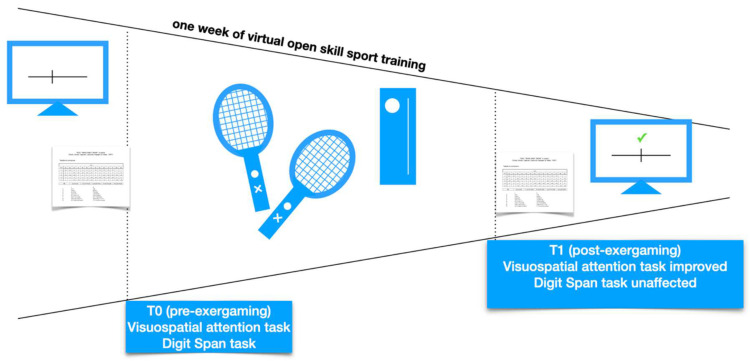
Schematic representation of experimental procedure in ExerGame group. Subjects performed Milner Landmark and digit span tasks at t0. Then, they played exergame tennis for one week, one hour a day. Eventually, they were re-evaluated performing the same tasks.

In order to avoid possible confounding effects due to uncontrolled training, subjects were asked to keep the condition of lack of physical and video gaming activities during the week preceding the first evaluation, when a visuospatial attention task and a control memory task were performed (t0). Subjects of the experimental groups then practised a tennis videogame one hour per day, for seven days, with an increasing level of difficulty automatically proposed by the game (except for the Sedentary group—not playing any video game and maintaining the lack of physical activity). This game time was arbitrarily chosen based on the student’s free time. At the end of the training (t1), the visuospatial and control memory tasks were repeated (Figure 1).

#### 2.2.1. Visuospatial Attention Task (Midline Judgment Task)

**Figure 2:**
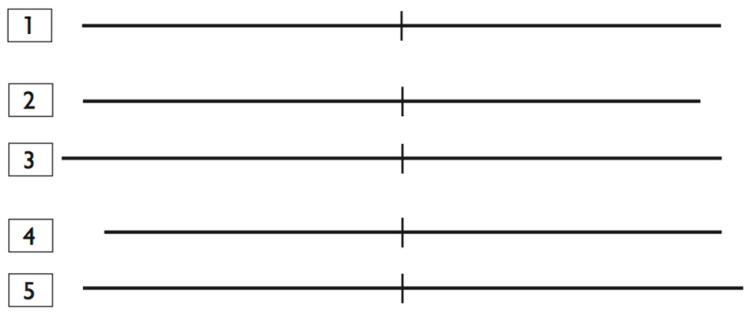
Visual stimuli presented to the subjects. For each stimulus, subjects made a forced choice decision of “equal”, “longer right” and “longer left”. Line 1: right segment, 75 mm; left segment, 75 mm (exactly bisected). Line 2: right segment, 70 mm; left segment, 75 mm (left elongated). Line 3: right segment, 75 mm; left segment, 80 mm (left elongated). Line 4: right segment, 75 mm; left segment, 70 mm (right elongated). Line 5: right segment, 80 mm; left segment, 75 mm (right elongated).

With respect to visuospatial attention tasks, a digital version of the modified Milner Landmark Task [34,35] was employed, a modified version of the line bisection task. This task has been diffusely employed in studies on visuospatial attention both in healthy subjects and in neglect patients [34,35,37]; here, it was chosen because it did not show any learning effect in previous reports [34]. Subjects were seated comfortably on a chair at reading distance (∼60 cm) in front of a 15” computer screen with a 100 Hz refresh rate. The subject’s seat was positioned so that their eye level was at the middle of the display monitor that was centred on his/her sagittal mid-plane. Visual stimuli were generated using Psyscope X software (version 77B, http://psy.cns.sissa.it, accessed on 15 May 2023) [38] and represented by black, 1 mm thick horizontal lines transected by a 1 mm thick and 1 cm long vertical bar, presented on a white background with the transector exactly coinciding with the centre of the screen. Five differently bisected lines—two left-elongated, two right-elongated and one centrally bisected (see Figure 2 for details)—were randomly presented for 50 ms on the computer screen. Before stimulus presentation, the subject was required to fixate on a central target (a black “*” character) that disappeared after 250 ms, as soon as the visual stimulus was flashed. Subjects were instructed to recognise the longer segment of the line with three possible responses—longer left, equally long, longer right—by pressing as soon as possible the corresponding key (V, B and N) marked with a coloured dot over a QWERTY Italian keyboard. Three blocks of 18 trials were presented (three repetitions for left or right elongated lines and six repetitions for exactly bisected lines, so that an equal number of left-elongated, right-elongated and exactly bisected lines were presented), for a total of 54 lines. A two-minute inter-blocks interval was observed. The whole task took about 10 min to complete. Reaction Times (RTs) and number of errors were recorded. According to previous studies [39,40], in order to evaluate laterality bias in attention control, we scored the performance of the subject on each trial as follows (described numbered lines are reported in Figure 2): 0 = correct response; 1 = right segment of line 1 judged longer, or left and right segments of lines 2 and 3 judged equal (rightward bias); −1 = left segment of line 1 judged longer, or left and right segments of lines 4 and 5 judged equal (leftward bias); 2 = right segment of lines 2 and 3 judged longer (rightward bias); −2 = left segment of lines 4 and 5 judged longer (leftward bias). 

#### 2.2.2. Control Memory Task

Regarding the control memory task, a short-term memory task was administered, the digit span forward task [41], which was performed using the standard method. This task is known to engage a phonological short-memory storage system, not related to spatial performance [41], and it was employed in order to exclude a generalised and unspecific effect on cognitive performance. The task was completed within about 5 min.

#### 2.2.3. Stimulation task

For the ExerGame group, the stimulation task was the tennis game included in the Wii^®^ sports software, with the participant playing tennis matches alone against the computer opponent. The video-game platform used was a Nintendo Wii^®^ (Nintendo^®^ Inc., Kyoto, Japan). The controller used was a Wii^®^ remote, a wireless three-axis accelerometer that works with gesture recognition. 

For the Non-ExerGame group, the stimulation task—similar to the first one but in a non-exergame platform—was Tennis World Tour (Nacon, Lesquin, France), with the participant playing tennis matches alone against the computer opponent. The video game platform used was a PlayStation 4^®^ (Sony^®^ Inc., Tokyo, Japan). The controller used was a Ps4 remote, a standard, wireless, hand-controlled device. 

Lastly, the Sedentary group was evaluated performing the same tasks at t0 and after a week (t1), without receiving any stimulation task in between, as control.

### 2.3. Statistical Analysis

Statistical analyses were performed using Statistica software (version 8.0; Dell Software, Tulsa, OK, USA, www.statsoft.com accessed on 15 May 2023) and significance levels were set at alpha = 5%.

Parametric tests were used since data resulted to be normally distributed (Shapiro–Wilk *p*-value > 0.05), according to previous literature [42]. Repeated measures (RM) ANOVA was used to compare the percentage of errors (number of wrong responses/total number of stimuli × 100) (as “within-subject factor”), and the percentage of leftward and rightward errors in the Pre- vs. Post-game conditions (as “within-subject factor”) were arcsine transformed and analysed by means of two-way ANOVA. Based on a previous study in which the same experimental apparatus and experimental methods were used [4], RTs higher than 1000 ms were excluded from the analysis as they were considered response errors. A paired *t*-test was used to compare digit span scores, as well as mean scores and RTs in the Pre- vs. Post-game conditions. 

## 3. Results

The results obtained on errors, scores, RTs and the digit span task are subdivided relative to each experimental group (EG, NEG and SE groups).

### 3.1. ExerGame group

#### 3.1.1. Midline Judgment Task

##### Errors

**Figure 3:**
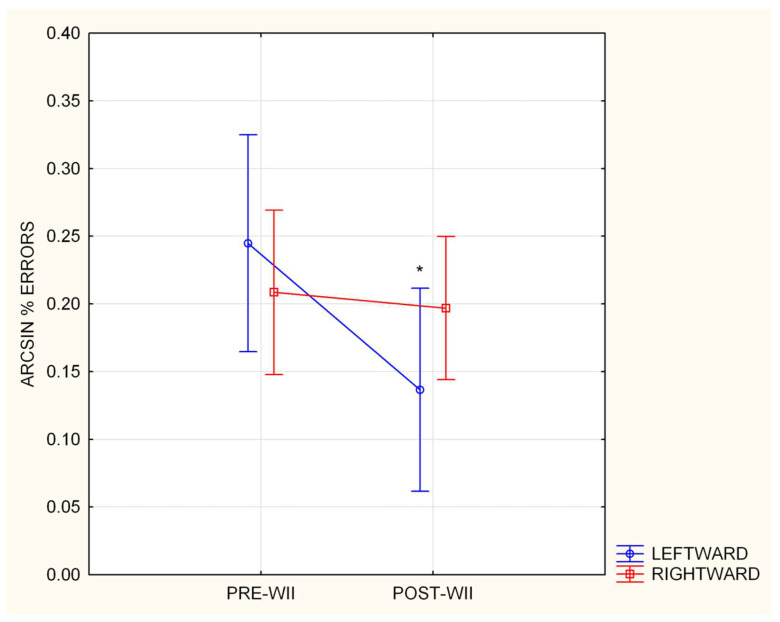
Arcsine transformation of the mean percentage of leftward and rightward errors in the Pre- and Post-game conditions in ExerGame group. Leftward pre-Wii vs. leftward post-Wii * *p* < 0.05. Error bars represent SE.

RM ANOVA on the percentage of errors showed a significant main effect of Pre- vs. Post-game conditions [(F (1, 14) = 7.24, *p* = 0.017, η*p*2 = 0.93] and the interaction of condition X side: [F (1, 14) = 5.48, *p* = 0.03, η*p*2 = 0.28]. As indicated in Figure 3, the Duncan post-hoc analysis on the arcsine transformation of the percentage of errors towards the left side was significantly decreased (*p* = 0.003), while there was no difference in the percentage of errors towards the right (*p* = 0.69), thus confirming the hypothesis of a specific effect on visuospatial attention isotropy. No other significance was found (Pre-game percentage of errors towards left-side vs. Pre-game percentage of errors towards right-side = 0.2; Post-game percentage of errors towards left-side vs. Post-game percentage of errors towards right-side = 0.57).

##### Scores

**Figure 4:**
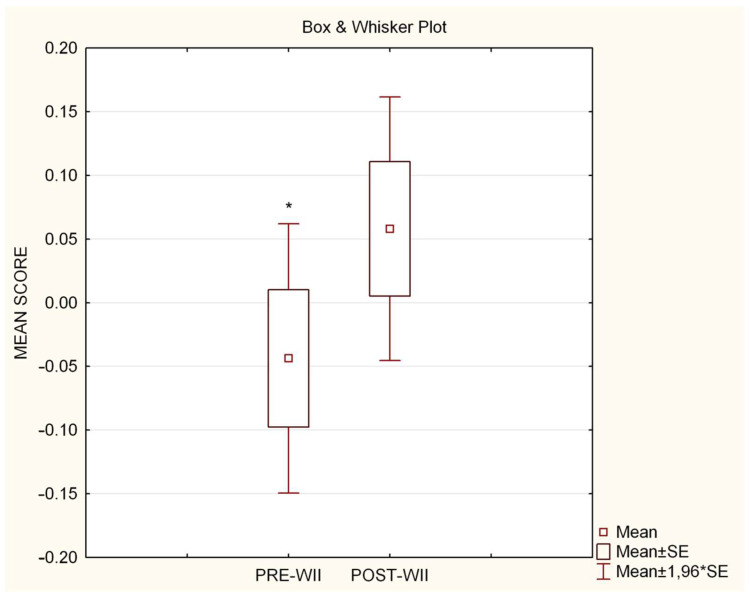
Mean scores in the Pre- and Post-game conditions. Positive values correspond to rightward responses and negative values to leftward responses in the ExerGame group. Pre-Wii vs. post-Wii * *p* < 0.05.

The *t*-test performed to compare the Pre- vs. Post-game scores showed a significant difference (*t* = −2.14, *p* = 0.050). Figure 4 depicts the mean scores; while the Pre-game condition subjects tend to perceive the left segment of the line as longer than the right (Pre-game mean score = −0.04, on a scale of −2/+2), the opposite pattern was found in the Post-game condition (Post-game mean score = 0.05, on a scale of −2/+2). This confirmed the pseudoneglect phenomenon in Pre-game condition, with slight reduction in the Post-game condition. 

##### Reaction Times

**Figure 5:**
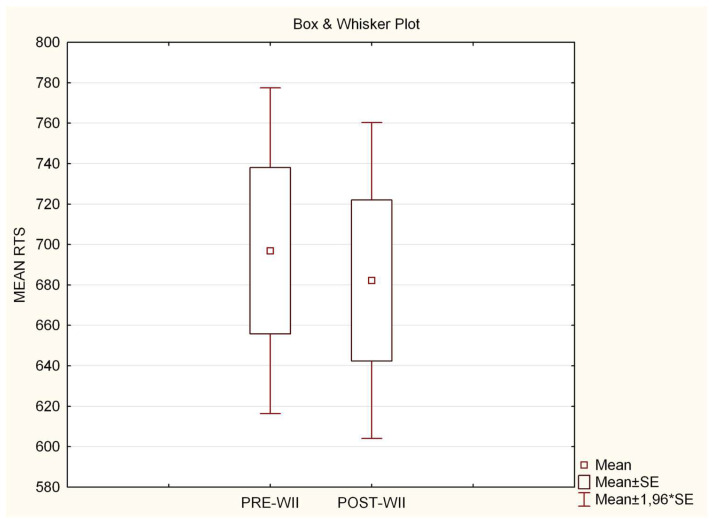
Mean Reaction Times in the Pre- and Post-game conditions in ExerGame group. * *p* < 0.05.

The *t*-test performed on RTs of correct answers comparing Pre- (mean RT: 696.91 +/− 159.17 SD) vs. Post-game (mean RT: 682.20 +/−154.35 SD) conditions did not show any significant changes in the speed of responses after the training (*t* = 0.26, *p* = 0.79), as depicted in Figure 5 representing the mean RTs.

#### 3.1.2. Digit Span Task

*t*-test for paired data did not show a significant difference in the Pre- (mean score: 5.64 ± 0.85 SD) vs. Post-game (mean score: 5.91 ± 0.92 SD) (*t* = −1.73, *p* = 0.1) conditions. 

### 3.2. Non-ExerGame Group

#### 3.2.1. Midline Judgment Task

##### Errors

The analysis on percentage of errors did not show any significant main effect of the Pre- vs. Post-game conditions [(F (1, 14) = 0.012, *p* = 0.91)], nor the interaction of condition X side: [F (1, 14) = 0.18, *p* = 0.67], thus showing a lack of any effect of traditional videogaming on visuospatial attention isotropy.

##### Scores

The Pre- vs. Post-game scores did not show any significant difference (*t* = −0.79, *p* = 0.43), with a Pre-game mean score of −0.09 and Post-game mean score of −0.06 on a scale of −2/+2. 

##### Reaction Times

RTs of correct answers comparing Pre- (mean RT: 698.28 +/−88.98 SD) vs. Post-game (mean RT: 669.08 +/− 66.10 SD) conditions did not show any significant changes (*t* = 1.36, *p* = 0.19) in the speed of responses after the training.

#### 3.2.2. Digit Span Task

Statistical analysis did not show a significant difference in the Pre- (mean score: 5.86 ± 0.68 SD) vs. Post-game (mean score: 6.06 ± 0.67 SD) (*t* = −1.87 *p* = 0.08) conditions.

### 3.3. Sedentary Group

#### 3.3.1. Midline Judgment Task

##### Errors

The percentage of errors did not show any statistical significance in terms of main effect of the Pre- vs. Post-game conditions [(F (1, 14) = 0.65, *p* = 0.43)], nor the interaction of condition X side: [F (1, 14) = 3.32, *p* = 0.08.], thus showing a lack of any effect of retest on visuospatial attention isotropy.

##### Scores

The Pre- vs. Post-game score did not show any significant difference (*t* = −1.44, *p* = 0.17), with a Pre-game mean score of −0.17 and Post-game mean score of −0.10, on a scale of −2/+2.

##### Reaction Times

RTs of correct answers comparing Pre- (mean RT: 718.92 +/− 95.32 SD) vs. Post-game (mean RT: 669.86 +/−67.15 SD) conditions did not show any statistical changes (*t* = 2.13, *p* = 0.051) in the speed of responses after the training.

#### 3.3.2. Digit Span Task

*t*-test for paired data did not show a significant difference in the Pre- (mean score: 5.73 ± 0.79 SD) vs. Post-game (mean score: 5.43 ± 0.59 SD) (*t* = 1.58, *p* = 0.1) conditions. 

## 4. Discussion

The present study investigated the role of active video gaming on the pseudoneglect phenomenon in healthy, non-athlete subjects. The main finding was the improvement in visuospatial attention isotropy due to exergame open-skill sport practice, likely counteracting the physiological imbalance in visuospatial attentional control. In line with the literature, here, a subtle [13,16] overestimation of the left side of bisected lines in Millner Landmark Test was found in the baseline score that slightly shifted to the right after practising with the open-skill exergame. Importantly, a marked leftward attentive bias was evidenced by the data on percentage of errors to the left in the pre-exergame condition that was counterbalanced in the post-exergame condition, as shown by the non-significant difference between leftward and rightward errors. The training seemed to be effective even after just one week, in line with other similar experiments of video game training [43]. A growing amount of evidence supports that cognitive performance can be improved by physical exercise, by means of increased metabolic activity, with selectively greater benefits for task components that require greater amounts of inhibitory control [44]. It could thus be argued that this could account for the results we obtained considering the described increased metabolic demands, even if small, due to exergames [19]. Conversely, the lack of effects on memory apparently rules out the possibility of unspecific improvements in cognitive functions. Moreover, a study by O’Leary and colleagues [45] showed that single bouts of exergames (as traditional video games) do not lead to any benefit in cognitive performance when compared to aerobic treadmill exercise. This is not surprising, considering the very low metabolic demand of an exergame compared with physical exercise. While it should be taken into account that the control task (digit span) can be globally less sensitive to a short training compared to the visuospatial task, it is worth noting that working memory was effectively trained in the study by Laine [46], who employed an even shorter (30’ single session) training. The data arising from the NEG group seem to suggest that the active gesture mimicry could play a crucial role in visuospatial remapping as we did not find any significant effects on this group, in which a traditional controller was used, since it seemed unable to improve visuospatial attention lateralisation. This finding can be interpreted as directly due to the type of interaction through gesture recognition or, alternatively, due to a higher engagement or to a more realistic experience. However, further experiments should be performed to better explore these hypotheses. Results from this study are consistent with other works comparing exergames, classic video games and control group, where exergame training was able to exert more significant cognitive and physical benefits than video game [41]. Differently from previous results collected from real, open-skill sport players [4], in the present study subjects did not become faster after the exergame sport training. Considering that RTs can also be affected by a pure motor output, this finding could be explained by the higher workload (3.37 ± 0.97 h/day) and duration of training (many years vs. 1 week) of volleyball players. 

As expected, the control group of sedentary people not playing any sport game did not show any changes in visuospatial performance, excluding any possible practice effect due to test–retest repetition. This is not surprising since the Milner Landmark task does not usually entail a significant learning effect when used in similar paradigms [34]. A possible effect of general arousal on pseudoneglect could also be considered as an alternative explanation to account for our data. However, in our results, the lack of effect on the memory task [47], where no different arousal level can be reasonably expected, seems to rule out potential nonspecific effects of arousal. A possible limitation of our study could be the absence of an exergame closed-skill sport group, which could have paralleled the results of real rowers, as in a previous work [4]. However, it was hard to find a closed-skill sport game that really lacks environmentally changing stimuli, maybe due to the intrinsic playful nature of exergames. As a matter of fact, the different spatial reference frame of the midline judgement task (allocentric) and the stimulation task (egocentric) could be questioned. Regarding this point, it is worth noting that it has been proven that the brain can remap one’s body representation in virtual reality to match what is shown in the simulation both in first person and in third person [48]. However, since pseudoneglect has been described in both egocentric and allocentric space mapping [14,16], it can be speculated that our stimulation task could be effective in modulating visuospatial performance in the midline judgement task. 

In open-skill sports, the environment is constantly changing, and movements must be continually adapted [49]. One could argue that people who are genetically advantaged in terms of visuospatial performance also have more chance to be selected for career progression in the field of open-skill sports. An alternative but not mutually exclusive hypothesis, as evidenced by the present results, is that daily training in an open-skill environment is able to modulate visuospatial attention function. Several papers have focused on attentional functions on athletes [50,51,52] but, to date, there is very little evidence of the possibility to modulate visuospatial attention lateralisation. The few data available are derived from neuro-pharmacological [53] or non-invasive brain stimulation studies in both negligent patients [54] and neurologically intact people [11,34,35,39], also hypothesising physiological bases of learning mechanisms [55]. Even if the second approach has less burden of side effects than the first, its availability, cost and experimental nature dramatically limit its use on the intriguing challenge of improving human cognitive performance. Recently, other “bottom-up” strategies have been developed such as optokinetic, transcutaneous electrical nerve stimulation and vestibular stimulation. Among these, neck muscle vibration (NMV) seems to be a very promising technology that can be easily integrated with other interventions [56]. On the other hand, the new generation of video game platforms are relatively cheap and easily available. Moreover, thanks to the fast advance in technology of gesture recognition, it is also possible to amplify weak movements, allowing physically impaired people to have similar gaming experiences to the average population [57]. Undoubtedly, active video gaming will represent a more adaptive tool for modulating higher processes and exploring their neural correlates, which could fit well from an integrative perspective with exogenous drug applications or brain stimulations in basic research approaches [58,59,60,61,62] or new interventions such as NMV. 

Further advantages of active video gaming comprise the fact that when using the tennis game of the exergames console, the player uses the entire upper limb to perform the task. The game also provides motivational features to encourage the user repetitively to improve their performance. Moreover, there is a growing knowledge on the safe use of active video gaming sports for rehabilitative purposes [26,27,63,64], and in older age [65], suggesting a benefit on neuronal homeostasis [66]. Effects of exergames on cognitive performance have been reported in different cognitive domains, such as attentional processing and executive functions [32,33]. Regarding the latter point, it is worth noting that stronger effects on specific subfunctions (reaction times in inhibition and switching) have been recently described [67]. Nevertheless, our work showed a specific effect on visuospatial attention lateralisation, which we speculate is related to the type (open-skill) of exergaming that parallels the effect of real, open-skill sport training rather than a nonspecific improvement in cognition, reflected by the lack of an effect on short-term memory. For this reason, we strongly support that an exergame open-skill sport environment could be useful not only to improve visuospatial attention in healthy subjects but also for rehabilitative purposes in people affected by neglect syndromes. Thus, exergame training might be a promising future strategy to improve cognitive and physical performance by active video gaming.

## 5. Conclusions

The present study falls within the framework of investigations relating to novel technological tools on physical and cognitive performance [33] in order to unveil active video gaming as a putative modulator of neural networks and to evaluate underlying cognitive processes. This could play a core role in the broader context of investigating the relationship between novel technological tools, physical activity and cognitive performance.

## Data Availability

Original data can be requested via mail: giuditta.gambino@unipa.it.
